# Deep learning-based robotic cloth manipulation applications: systematic review, challenges and opportunities for physical AI

**DOI:** 10.3389/frobt.2026.1752914

**Published:** 2026-02-06

**Authors:** Ningquan Gu, Mitsuhiro Hayashibe, Kyo Kutsuzawa, Hui Yu

**Affiliations:** 1 Neuro-Robotics Lab, Department of Robotics, Graduate School of Engineering, Tohoku University, Sendai, Japan; 2 Graduate School of Science and Engineering, Saitama University, Saitama, Japan; 3 School of Psychology & Neuroscience, University of Glasgow, Glasgow, United Kingdom

**Keywords:** cloth unfolding and folding, deep learning, LLM, physical AI, robotic manipulation, systematic review

## Abstract

Cloth unfolding and folding are fundamental tasks in autonomous robotic cloth manipulation as Physical AI. Driven by recent advances in deep learning, this area has developed rapidly in recent years. This review aims to systematically identify and summarize current progress in deep learning-based cloth unfolding and folding. Following the Systematic Reviews and Meta-Analyses (PRISMA) guidelines, 41 relevant papers from 2019 to 2024 were selected for analysis. We examines various factors influencing cloth manipulation and find that, while current methods show impressive performance, several challenges remain unaddressed. These challenges include irregular cloth sizes and diverse initial garment states. Concerning datasets, there is a need for improved real-world data collection systems and more realistic cloth simulators, and the Sim2Real gap must be carefully considered. Additionally, the review highlights the importance of incorporating multi-modal sensors into current platforms and the emergence of novel primitive actions that enhance performance. The need for more consistent comparison metrics is emphasized, and strategies for addressing failure modes are discussed to further advance the field. From an algorithmic perspective, we reorganize existing learning methods into six learning and control paradigms: perception-guided heuristics, goal-conditioned manipulation policies, predictive and model-based state representation methods, reward-driven reinforcement learning over primitive actions, demonstration-driven skill transfer methods, and emerging large language model-based planning methods. We discuss how each paradigm contributes to unfolding and folding, their respective strengths and limitations, and the open problems that arise. Finally, we summarize the remaining challenges and provide future perspectives for physical AI.

## 1 Introduction

Cloth manipulation is essential in daily life and various industries. Automating this process has significant implications for improving quality of life and enhancing productivity and efficiency in laundry services, retail, and manufacturing. However, cloth manipulation presents challenges for robotics due to the infinite-dimensional configuration space, self-occlusion, and the complex dynamics of cloth. Robotic cloth manipulation encompasses various operations, such as cloth unfolding and folding (Seita et al., 2019; Tsurumine et al., 2019; Zhou et al., 2024; Yang et al., 2024), assisted human dressing (Zhang and Demiris, 2020), ironing (Li et al., 2016), and sewing (Ku et al., 2023). Among these, folding and unfolding operations are the most fundamental tasks. They are crucial for applications such as laundry automation, retail inventory management, and personal assistant robots, playing a significant role in both everyday life and industrial processes. Maitin-Shepard et al. (2010) developed the first system using a PR2 robot to unfold and fold wrinkled towels. However, early methods for unfolding or folding were often slow and lacked the ability to generalize to arbitrary initial and target states.

In recent years, researchers have explored deep learning-based (DL-based) approaches for cloth manipulation, which have shown improved results compared to traditional methods. For instance, Seita et al. (2019) utilized the YOLO detection algorithm to identify keypoints on a blanket, facilitating the unfolding task and demonstrating the efficacy of DL-based methods. Later Canberk et al. (2023) employed deep reinforcement learning (RL) to perform garment unfolding, ironing, and folding tasks. The application of DL-based methods has not only introduced algorithmic advancements but also impacted other elements, such as datasets, manipulation strategies, and comparison metrics.

In this systematic review, we examine recent advances in DL-based robotic cloth unfolding and folding. Prior surveys have discussed related aspects such as cloth perception for assistive manipulation (Jiménez and Torras, 2020) and deformable-object modeling (Hou et al., 2019). Nocentini et al. (2022) reviewed learning-based cloth manipulation and dressing from a supervision-type perspective (e.g., supervised, reinforcement, imitation learning), but their coverage ends in 2019 and therefore does not reflect the substantial methodological progress made in recent years. Moreover, supervision-based taxonomies (Nocentini et al., 2022) provide a conventional perspective but do not adequately capture the underlying perception, representation, and control structures in the cloth-manipulation field.

To address this gap, we reorganize the literature into six learning-and-control paradigms that more directly reflect how existing methods perceive cloth, represent its state, and decide actions. Our review focuses on DL-based approaches for cloth unfolding and folding under this paradigm-oriented perspective.

To clarify the scope of our review, we briefly characterize the two core tasks considered in this survey: cloth unfolding and cloth folding.

The unfolding process consists of applying a sequence of actions to transform a cloth from an arbitrary crumpled configuration into a flattened state with maximal coverage. This process typically exhibits the following characteristics:

- Random Initial State: The starting configuration of the cloth can vary significantly, often being crumpled in an arbitrary manner.
- Various Manipulation Strategies: The manipulation strategies are various, e.g., one or multiple robot arms, diverse action primitives, combination of actions.
- Uniform End Criterion: Coverage is the primary criterion for the unfolded result. Further, other customized configurations, such as cloth orientation, are also considered in some cases.

In contrast, the folding process starts from an unfolded or nearly unfolded configuration and aims to reach a predefined structured goal shape. Its key characteristics include:

- Regular Initial and Goal State: The starting configuration of the cloth is flattened, with variable positions and sizes. The folding goal state is predefined.

We reviewed 41 eligible papers published between 2019 and 2024, analyzing their task contents, datasets, platforms, primitive actions, evaluation metrics, failure modes, and learning methodologies. As DL-based techniques advance, these related aspects continue to evolve in parallel. Despite notable progress, significant challenges remain, leaving ample opportunities for future research.

The systematic review makes the following contributions:

1. A paradigm-oriented taxonomy: We reorganize recent DL-based cloth manipulation methods into six learning-and-control paradigms that more accurately reflect their perception, representation, and decision-making structures, providing a more meaningful alternative to supervision-based taxonomies used in prior surveys.
2. A comprehensive analysis of unfolding and folding tasks: We clearly define and distinguish cloth unfolding and folding processes, and analyze key aspects including task contents, datasets, manipulation platforms, primitive actions, metrics, and common failure modes.
3. Insights into challenges and future opportunities: We identify the limitations across paradigms, and outline promising directions for advancing DL-based cloth manipulation.

The remainder of this paper is structured as follows. Section 2 outlines the methodology used to identify relevant literature on DL-based cloth unfolding and folding, detailing the criteria for paper inclusion and exclusion. Section 3 presents the outcomes of the literature search from seven aspects. Section 4 discusses these findings and current challenges while suggesting potential solutions for future research. Finally, Section 5 provides a conclusion to the review.

## 2 Methods

The method employed to identify relevant empirical papers follows the guidelines of the Preferred Reporting Items for Systematic Reviews and Meta-Analyses (PRISMA) (Liberati et al., 2009). This review focuses on DL-based cloth manipulation, specifically cloth unfolding and folding, within the realm of robotic manipulation. It encompasses a thorough examination of empirical papers published between 2019 and 2024, aiming to uncover the latest research developments and trends in this field. The search terms and their combinations were defined as follows:

(“cloth” OR “fabric” OR “garment” OR “towel” OR “textile” OR “blanket”) AND (“robot*”) AND (“shape*” OR “unfold*” OR “fold*” OR “smooth*” OR “flatten*”) AND (“learning based” OR “learning-based” OR “deep learning” OR “deep-learning” OR “neural network” OR “reinforcement learning” OR “RL” OR “SL” OR “imitation learning” OR “IL” OR “supervised learning”)

The rationale behind the selection of specific search terms and their combinations is outlined as follows:

- Domain-Specific Keywords: To capture all pertinent aspects of cloth and textile manipulation, terms related to various cloth types (e.g., “cloth”, “fabric”, “garment” etc.) and actions (e.g., “shape”, “unfold”, “fold” etc.) were included.
- Robotic Related: The inclusion of the term “robot*” ensures the search is specifically focused on robotic system.
- Comprehensive and Inclusive Search: Synonyms and variations of core terms related to DL methodologies (e.g., “learning-based”, “deep learning”, “neural network” etc.) were included to cover the wide spectrum of terminologies used across different studies.
- Simultaneous Inclusion Requirement: The empirical robotic manipulation papers should include all three categories of terms simultaneously to ensure comprehensive coverage of the topic.

**FIGURE 1:**
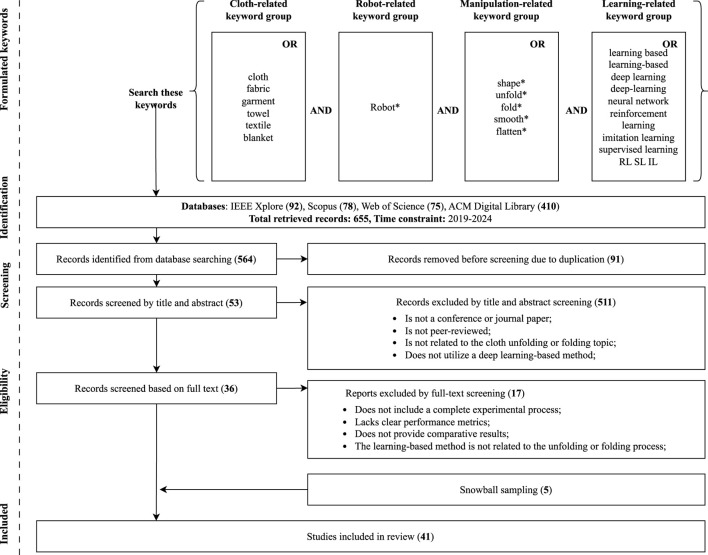
Flowchat of the literature selection process.

See Figure 1, a bibliography was developed based on searches in IEEE Xplore, Scopus, Web of Science, and ACM Digital Library between 2019 and 2024. We collected 655 related records from these four databases, after excluding duplicates, screened based on abstract and full text, 36 records remained. To make the research sample for the review more comprehensive, we employed backward snowball sampling (Jalali and Wohlin, 2012) with the same exclusion criteria. At the end of the search, 41 papers were identified for our systematic review. More details are provided in the eligibility stage in Figure 1. We also find that most eligible studies were published in conferences (63.4%).

## 3 Synthesis of results

**FIGURE 2:**
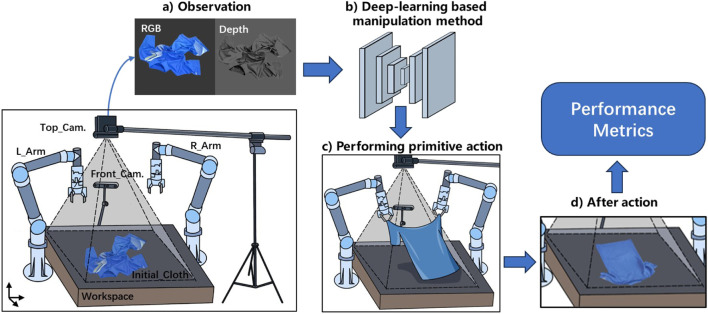
Overview of robotic cloth unfolding and folding manipulation process. The robotic manipulation platform is built with robot arms, a top-view camera for main observation, and an optional front-view camera. **(a)** During manipulation, the main top-view camera observes the current state of the cloth to provide the RGB or depth image. **(b)** After inputting the image into the DL algorithm, it perceives the scene and plans the manipulation action. **(c)** The robot executes the planned manipulation with primitive actions. **(d)** The cloth is manipulated into a new state. The process will continue iteratively until the cloth meets the termination criteria and evaluates according to performance metrics.

We illustrate the cloth unfolding and folding manipulation process in Figure 2. In this section, we synthesize the eligible papers by examining various important aspects during the manipulation process. These aspects include task contents, datasets, manipulation platforms, primitive actions, performance metrics, failure modes, and learning methods.

### 3.1 Task contents

Cloth unfolding and folding are closely related processes, with unfolding often serving as a preliminary step for folding. Although they are sometimes treated as distinct tasks, they are closely connected. Table 1 summarizes the distribution of tasks and manipulated object types in the 41 reviewed papers. Overall, most studies focus on a single task and primarily use small towels or napkins, while only a minority address large cloths or more complex garments.

**TABLE 1:** Summary of task contents in the 41 reviewed papers.

Category	Count (%)
Task type	
Folding/unfolding only	29 (70.7%)
Both unfolding and folding	12 (29.3%)
Manipulated object type	
Towels/napkins (within reach)	22 (53.7%)
Garments (T-shirts, skirts, trousers)	12 (29.3%)
Both towels and garments	4 (9.8%)
Large cloths (beyond reach)	3 (7.3%)

### 3.2 Datasets

Data plays a crucial role in the development of DL-based methods (Tampuu et al., 2020) for cloth unfolding and folding. Due to the complexity of robotic manipulation of deformable objects (Zhu et al., 2022), which includes various cloth configurations, interactions with physical environments, diverse robot capabilities and morphologies, and different hardware setups across labs, there is currently no universally recognized public dataset in this field. Consequently, each of the 41 eligible papers in this review has collected its own dataset. Dataset collection was primarily conducted through two strategies: simulation and real-world experiments.

#### 3.2.1 Types of collected data

Due to differences in learning paradigms, the types of data collected vary across studies. Visual information, such as RGB images, depth images, and grayscale images, is collected in all selected papers. In addition to visual observations, other data modalities are also used to provide richer supervision. These include keypoint annotations (Seita et al., 2019; Hoque et al., 2022b) for keypoint perception, action-related data such as pick and place points (Tsurumine et al., 2019), pick-and-pull directions (Seita et al., 2020; Hoque et al., 2022a), and manipulation trajectories (Chen et al., 2022) for supervising control policy learning, as well as manipulation stage or phase annotations (Mo et al., 2022; Wang et al., 2022) to support temporal decomposition of manipulation processes. Another category of collected data is cloth state representations, such as cloth particle poses (Weng et al., 2022) and cloth mesh representations (Tanaka et al., 2021; Ma et al., 2022), which allow explicit representations of cloth dynamics and deformation. A single study may collect multiple types of data depending on its learning formulation.

#### 3.2.2 Dataset collection from simulation

Collecting datasets or training in simulation is a widely used strategy for learning-based robotic cloth manipulation (65.9%, 27 out of 41). The most commonly used simulators in this field include gym-based environments, such as SoftGym (Lin et al., 2021), which accounts for more than half of the simulation studies, and FEM-based simulators integrated with Gym (Seita et al., 2020), as well as Blender, MuJoCo, and others. SoftGym (Lin et al., 2021), built on the PyFleX (Li et al., 2019) bindings to NVIDIA FleX, can load various garment meshes including T-shirts, trousers, and dresses. However, its current version does not support loading robot models due to NVIDIA’s permission constraints, limiting its use for training real robots that rely on Cartesian control. FEM-based fabric simulators interfaced with OpenAI Gym provide another option, though the authors acknowledge that these simulators exhibit lower physical fidelity compared to other engines. Hietala et al. (2022) instead used MuJoCo, which supports loading robot URDF models. All simulation datasets mentioned above were collected entirely within simulated environments. Xue et al. (2023) employed RFUniverse (Fu H. et al., 2023), which enables the acquisition of cloth-environment interaction data through a Virtual Reality (VR) setup, thereby enhancing interactive capabilities from the real world to the simulated environment.

While simulation facilitates the development of DL-based methods, the gap between simulation and the real world remains a challenge in robotic cloth manipulation. This gap mainly comes from inaccurate modeling of cloth properties, limited visual diversity, dynamics mismatch, and unmodeled interactions, etc. Therefore, addressing the Sim2Real gap is an important consideration (Collins et al., 2019; Matas et al., 2018). The strategies include Domain Randomization (DR), Data Augmentation (DA), depth or point-cloud observations, fine-tuning with real-world data, texture replacement, and training under real settings, and others. Table 2 summarizes the primary Sim2Real gaps encountered in cloth manipulation and the corresponding strategies used to mitigate them.

**TABLE 2:** Summary of Sim2Real gaps in robotic cloth manipulation and the corresponding mitigation strategies.

Primary Sim2Real gap	Sim2Real strategy used
Inaccurate or oversimplified modeling of cloth physical properties and appearance variability across real garments. (Hietala et al., 2022; Blanco-Mulero et al., 2023; Seita et al., 2020)	DR: Randomizing cloth stiffness, mass, size, color, shading, lighting, background, and camera pose to improve robustness across fabric types
Limited visual diversity and viewpoint mismatch between simulated and real cloth observations. (Tanaka et al., 2021)	DA: Applying transformations such as cropping, rotation, flipping, and noise injection to enhance robustness and generalization
Photometric inconsistencies caused by lighting, texture, wrinkles, and shading in RGB images. (Weng et al., 2022; Mo et al., 2022; Ma et al., 2022)	Using depth/Point clouds: Using depth maps or point-cloud observations to reduce the photometric gap between simulation and reality
Residual dynamics mismatch and unmodeled interactions (e.g., friction, contact, grasping errors) after simulation pre-training. (Ha and Song, 2022; Gu et al., 2024)	Fine-tuning: Training policies in simulation and then fine-tuning them with real-world data
Visual domain gap arising from complex real-world textures and background clutter. (Xu et al., 2022)	Texture replacement: Replacing cloth and background textures in real images with simulation-like uniform colors
Mismatch between simulated controllers, sensors, or object properties and real robotic hardware. (Hietala et al., 2022; Wang et al., 2022)	Training with real settings: Incorporating real hardware characteristics or real-object textures directly into the simulation environment
Others: Task-specific data or model dependency, action and reward modeling gap. (Ganapathi et al., 2021; Ma et al., 2022; Canberk et al., 2023)	Geometric structure from visual representations, simplified action models, or specialized reward designs to facilitate Sim2Real transfer

#### 3.2.3 Dataset collection from real world

Collecting datasets or training in the real world presents challenges but offers significant benefits. It avoids the Sim2Real problem and generally results in better generalization compared to simulation-based training. However, the considerable workload of human annotation and the wear and tear on robots are notable factors. To reduce the labeling workload, Seita et al. (2019) proposed a color-based keypoint annotation strategy that enables automatic extraction of cloth corners from color-marked visual observations for depth images. Later, Fu T. et al. (2023) adopted a similar color-labeling strategy for dataset collection and segmentation training, using different paint color for cloth corners and edges. However, the training dataset collected using this approach includes only depth images, which consist of a single depth channel and contain no color cues. Seita et al. (2020) reported that the color contrast between the fabric and the workspace in RGB images can facilitate better performance. Furthermore, depth sensing requires dedicated hardware. Therefore, Thananjeyan et al. (2022) addressed this by introducing a UV-based labeling technique for deformable objects in RGB images, referred to as Labels from UltraViolet (LUV). Transparent UV fluorescent paint is invisible under standard light but detectable under UV light. Similarly, Gu et al. (2024) adopted this method during their finetuning process.

**FIGURE 3:**
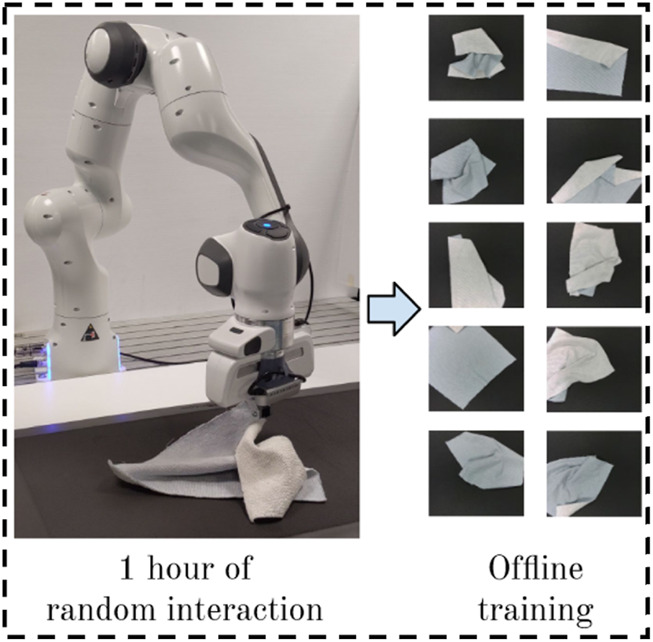
Collecting an hour of experience dataset on the real robot (PMLR image credit Lee et al., 2021, licensed under CC BY).

The color-labeling methods mentioned above can efficiently provide keypoints for cloth manipulation. However, they may be inadequate in certain scenarios, particularly when RL or imitation learning is employed. Consequently, other data collection methods have been explored. For example, Hoque et al. (2022b) developed open-source software that enables humans to remotely control a robot for interacting with cloth and collecting demonstrations. Avigal et al. (2022) first annotated images with primitive actions and corresponding gripper positions and then trained a neural network to iteratively collect self-supervised data. Lee et al. (2021) required only 1 hour of random interactions with the cloth to develop their offline RL approach, which effectively handles complex sequential cloth folding (see Figure 3). In the new work of Lee et al. (2024), they further explored dataset collection by tackling the movement in human manipulation videos, making the dataset collection process more efficient and simpler.

### 3.3 Manipulation platforms

Robotic cloth manipulation platforms typically consist of two key components: the manipulation system and the vision system. The robot executes physical interactions with the cloth, whereas the vision module provides the perceptual observations required for state estimation and policy learning. This section summarizes the platforms adopted across the reviewed studies.

#### 3.3.1 Robot types

Most works employ single-arm manipulation. Specifically, 65.9% (27 out of 41) of the reviewed papers use a single robotic arm, while 31.7% (13 out of 41) adopt a dual-arm system. Only one study (Xu et al., 2022) utilizes a three-arm setup. Regarding robot brands, the Universal Robots series (particularly UR5) is the most frequently used in real-world experiments, followed by the Franka Emika Panda arm. For dual-arm settings, existing solutions either (i) combine two independent single-arm robots into a coordinated dual-arm system (Ha and Song, 2022; Gu et al., 2024; Xue et al., 2023) or (ii) rely on dedicated dual-arm robot platforms such as ABB YuMi (Avigal et al., 2022), Kawada HIRO (Tanaka et al., 2021), or PR2 (Wu Y. et al., 2020).

#### 3.3.2 Vision sensors

The vision observation types include RGB, depth, RGB-D, grayscale, and point cloud images. We systematically analyze these inputs, which are used for training or inference, as shown in Table 3. RGB information is the most commonly used observation. Hoque et al. (2022a) compared the results of RGB, RGB-D, and depth, and suggested that RGB-D provides the best performance for their visual foresight task. Lin et al. (2022) demonstrated that RGB-related information are sensitive to camera views and visual features, which also poses challenges in the Sim2Real transformation. Later, Weng et al. (2022) utilized depth images as their policy input because they found that using depth images or point clouds as the training dataset could minimize the gap between simulation and reality.

**TABLE 3:** Observation modalities used.

Observation type	Count (%)
RGB	22 (53.7%)
RGB-D	6 (14.6%)
Depth/point cloud	11 (26.8%)
Gray	1 (2.4%)
Depth and gray	1 (2.4%)

### 3.4 Primitive actions

The primitive actions used in cloth unfolding and folding manipulation include quasi-static pick-and-place (P&P), dynamic fling action, drag and mop, and air blowing. The manipulations described in the papers either use one or a combination of these four primitive actions.

#### 3.4.1 Pick and place action

The P&P configuration involves selecting a pick coordinate and a place coordinate. Initially, a robot grasps the cloth at the pick coordinate and lifts it to a certain height. The robot then moves above the place coordinate and finally places the cloth down and releases its grip. However, the P&P primitive action has its development. For example, Hoque et al. (2022a) provide pixel coordinates for the pick and place points, which the robot then uses to execute the action. Kase et al. (2022) decomposed and labeled the P&P action into finer phases: approach, grasp, pull, fold, release, and standby, to facilitate their cloth manipulation task. Avigal et al. (2022) incorporated the grasp angle into their manipulation process, estimating a pixel-wise value map for each gripper z-axis rotation to enhance the reliability of their P&P action. To achieve better performance, Blanco-Mulero et al. (2023) proposed a policy that optimizes parameters such as motion velocity and height within the P&P primitive action. Hietala et al. (2022) demonstrated that closed-loop feedback with parameterized P&P primitives significantly enhances adaptability in cloth manipulation, showing promise for more general and adaptive skills. Although the P&P action has been successfully utilized in cloth manipulation, it is relatively slow and constrained by the robot’s workspace.

#### 3.4.2 Dynamic fling action

**FIGURE 4:**
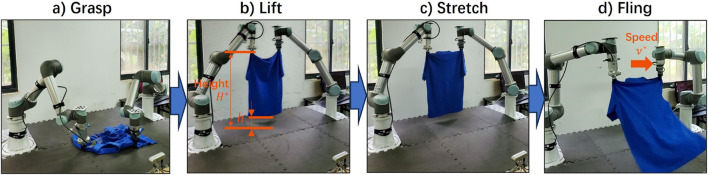
Process of flinging the garment to expand the coverage (Reprinted with permission from Gu et al., 2024, copyright 2024 IEEE). **(a)** Grasp **(b)** Lift **(c)** stretch **(d)** Fling.

To overcome the drawbacks of P&P actions, Ha and Song (2022) proposed a dynamic fling primitive that leverages object momentum for efficient unfolding. Their approach learns grasp locations while relying on predefined motion parameters, which may limit robustness across different cloth geometries or sizes. To address this issue, Gu et al. (2024) correlated the lift height with cloth size and adjusted the fling speed based on the height, which improved performance, as illustrated in Figure 4. Chen et al. (2022) further focused on learning fling action trajectories for garment unfolding using one robot arm, rather than a fixed fling trajectory.

Although the fling action can significantly expand the coverage, it is a coarse-grained manipulation method and is insufficient for fine-grained manipulation like P&P. Therefore, Canberk et al. (2023) strategically choose between the fling and P&P actions to efficiently and precisely unfold the garment. Their policy first utilizes the fling action to bring the cloth to a considerably unfolded state, then employs the P&P action to further expand it.

#### 3.4.3 Drag and mop action

During cloth manipulation, predicted manipulation points may be outside the robot’s reachable workspace or correspond to difficult-to-handle cloth configurations when using previous primitive actions. Therefore, Avigal et al. (2022) and Xue et al. (2023) introduce a drag action in the unfolding stage. This action involves two robots simultaneously dragging the cloth away from its center. By exploiting the friction between the cloth and the supporting surface, the drag action helps smooth out corners and wrinkles, such as sleeves trapped underneath the garment, and can also be used to reposition the cloth. For the folding stage, Xue et al. (2023) further introduce a related action primitive mop. Similar to drag, mop is used to adjust the cloth position when the predicted grasp or placement points are unreachable during folding. Another form of dragging is described by He et al. (2023), in which two robotic arms are used asymmetrically: one arm grasps the cloth and remains stationary, while the other drags the cloth away by a predefined distance. This strategy is particularly effective for handling long sleeves that are covered by or folded inside a T-shirt.

#### 3.4.4 Air blow action

The primitive actions described above manipulate the cloth either through sparse contact or by utilizing high speed robot. From the perspectives of dense force application and safety, Xu et al. (2022) proposed an air blow primitive action. In this approach, two robot arms grasp two points on the cloth, while a third arm operates a blower to apply air, expanding and unfolding the cloth. This action allows for the application of dense air forces on areas not in direct contact with the robot, thereby extending the system’s reach and enabling safe, high-speed interactions.

### 3.5 Performance metrics

For the unfolding task, almost all of the eligible papers use cloth coverage as their primary performance metric. The second most common metric is the number of action steps, which evaluates the efficiency of the unfolding policy. Time-related metrics, such as execution time and the time taken to determine actions, are also considered. Additionally, metrics like reward after actions (Canberk et al., 2023), cloth orientation (Gu et al., 2024), and MIoU (Xue et al., 2023) are used to evaluate unfolding performance.

In the folding task, the most commonly used metric is the folding success rate. However, the criteria for evaluating success vary. Some studies (Tanaka et al., 2021; Weng et al., 2022) use MIoU as their success rate metric. Lee et al. (2021) argue that the self-occluding, deformable nature of the cloth and the difficulty of observing a 3D state in a 2D image make it challenging to apply a quantitative MIoU metric. They introduced “visually consistent with the target image” as their success rate metric. Conversely, some papers (Ma et al., 2022; Hoque et al., 2022a) evaluate performance using the cloth particle distance between the goal and the result in the simulation, although this criterion cannot be used in the real world. Additionally, metrics such as inference and execution time (Avigal et al., 2022) are considered to compare efficiency. Wrinkle penalties (Hoque et al., 2022b) and normalized metrics (Chen and Rojas, 2024) are also used.

### 3.6 Failure modes

In cloth unfolding and folding tasks, frequent failure modes, in addition to common motion planning errors (Gu et al., 2024), include issues such as failing to grasp (Blanco-Mulero et al., 2023), incorrect numbers of grasped cloth layers (Fu T. et al., 2023; Xue et al., 2023; Xu et al., 2022; Ganapathi et al., 2021), losing grip (Avigal et al., 2022), and failed releases (Shehawy et al., 2023). Among these, multi-layer grasping is particularly influential as a failure mode. Other issues include inaccurate predictions (Wang et al., 2022) and the gap between simulation and reality.

### 3.7 Learning and control paradigms for cloth manipulation

Prior survey work (Nocentini et al., 2022) categorizes learning-based cloth manipulation methods according to supervision type: supervised learning (SL), unsupervised learning (USL), reinforcement learning (RL), and imitation learning (IL). While this perspective is too general as a machine-learning taxonomy and too coarse to reveal the algorithmic structures in cloth manipulation tasks.

**FIGURE 5:**
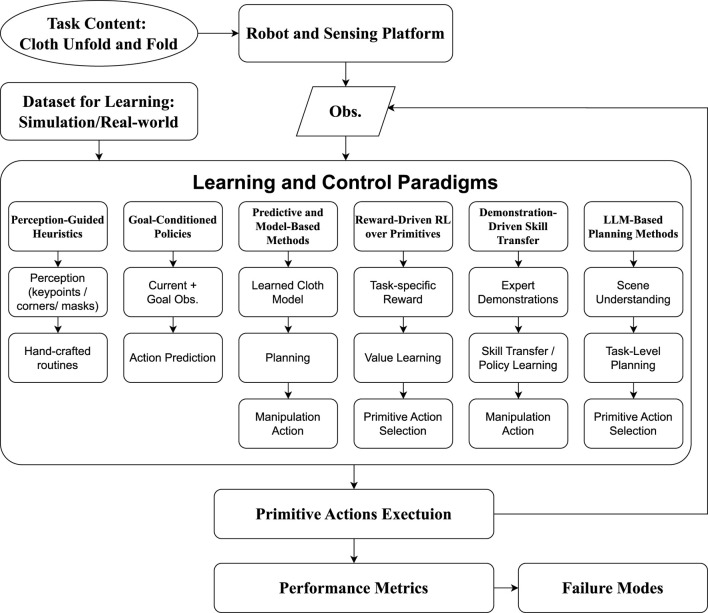
Conceptual overview diagram of the six learning and control paradigms in the robotic cloth unfolding and folding pipeline.

To better characterize how existing approaches perceive, represent, and act in cloth manipulation, we reorganize the eligible papers into six learning-and-control paradigms (Table 4) that more directly reflect their underlying design principles and highlight differences in perception requirements, control structures, and generalization strategies across cloth unfolding and folding tasks, offering a more fine-grained view. Figure 5 provides a conceptual overview of the learning and control paradigms within the overall robotic cloth unfolding and folding workflow discussed in this review.

- Perception-Guided Heuristic Methods: Vision networks (e.g., keypoint detectors or segmentation models) predict regions or contours (e.g., corners, masks, keypoints), which then feed into hand-crafted unfolding or folding routines.
- Goal-Conditioned Manipulation Policies: Policies that take the current observation and a desired goal configuration as input and output manipulation actions to reach the goal.
- Predictive and Model-Based State Representation Methods: Approaches that learn explicit cloth dynamics, latent state representations, or visuospatial models and use them for planning or control.
- Reward-Driven Reinforcement Learning over Primitive Actions: RL methods that optimize value or policy functions over discrete or continuous manipulation primitives using task-specific reward signals.
- Demonstration-Driven Skill Transfer Methods: Methods that learn manipulation policies primarily from expert demonstrations (simulation rollouts, robot teleoperation data, or human videos) and adapt them to the current situation.
- Large Language Model-Based Planning Methods: Approaches that leverage large language models (LLMs) or vision-language models (VLMs) to extract high-level semantic information from textual descriptions or visual observations, propose manipulation primitives, infer sub-goals, and generate high-level action plans.

**TABLE 4:** Statistics of the six learning-and-control paradigms across cloth unfolding and folding tasks.

Paradigm	Unfold (Count + refs.)	Fold (Count + refs.)
Perception-H	2: (Seita et al., 2019; Hoque et al., 2022b)	3: (Canberk et al., 2023; Gu et al., 2024; He et al., 2023)
Goal-cond	0: –	3: (Tanaka et al., 2021; Weng et al., 2022; Mo et al., 2022)
Predict.-model	7: (Ganapathi et al., 2021; Ma et al., 2022; Hoque et al., 2022a; Lin et al., 2022; Deng et al., 2023; Kadi and Terzić, 2024; Wu et al., 2024)	8: (Ganapathi et al., 2021; Ma et al., 2022; Hoque et al., 2022a; Cao et al., 2023; Deng et al., 2023; Zhou et al., 2024; Longhini et al., 2024; Wu et al., 2024)
Reward-driven	11: (Tsurumine et al., 2019; Ha and Song, 2022; Chen et al., 2022; Xu et al., 2022; Hoque et al., 2022b; Avigal et al., 2022; Canberk et al., 2023; Gu et al., 2024; Blanco-Mulero et al., 2023; Shehawy et al., 2023; He et al., 2023)	6: (Wu et al., 2020b; Lee et al., 2021; Salhotra et al., 2022; Hietala et al., 2022; Shehawy et al., 2023; Chen and Rojas, 2024)
Demo.-driven	7: (Seita et al., 2020; Hoque et al., 2022b; Xue et al., 2023; Fu et al., 2023b; Lee et al., 2024; Galassi et al., 2024; Yang et al., 2024)	6: (Wang et al., 2022; Hoque et al., 2022b; Kase et al., 2022; Tsurumine and Matsubara, 2022; Xue et al., 2023; Lee et al., 2024)
LLM-based	2: (Fu et al., 2024; Raval et al., 2024)	1: (Raval et al., 2024)

#### 3.7.1 Perception-guided heuristic methods

**FIGURE 6:**
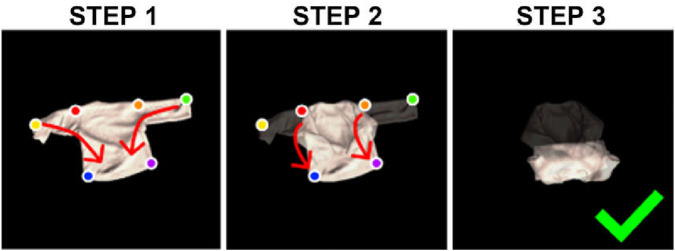
Keypoint-based heuristic folding method (arXiv image credit: Canberk et al. (2022), licensed under CC BY).

Early work on cloth manipulation often relies on explicit perception outputs, such as corners, edges, or segmentation masks, which are then mapped to hand-designed manipulation routines to realize simple folding or unfolding. Seita et al. (2019) used YOLO (Redmon et al., 2016) to detect blanket corners from depth images. The detected keypoints are passed to a keypoint-based heuristic controller: the robot grasps these points and pulls the blanket to increase coverage. Real-world experiments with two mobile manipulators and three blankets demonstrated strong generalization of this perception-guided heuristic pipeline. Several works adopt a similar structure for folding tasks, as illustrated in Figure 6. Canberk et al. (2023); He et al. (2023); Gu et al. (2024) use segmentation networks such as U-Net (Ronneberger et al., 2015), DeeplabV3 (Chen et al., 2017), and DeeplabV3+ (Chen et al., 2018) to localize keypoints or regions on flattened garments to fold the cloth.

#### 3.7.2 Goal-conditioned manipulation policies

**FIGURE 7:**
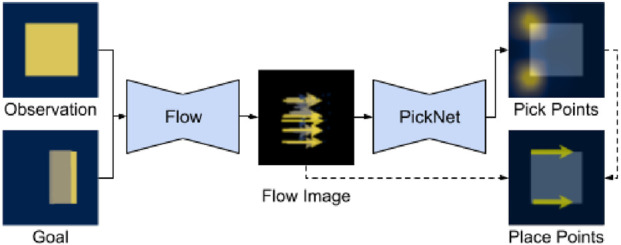
FabricFlowNet, a type of Goal-Conditioned Manipulation Policy (PMLR image credit: Weng et al. (2022), licensed under CC BY).

While perception-guided heuristics are effective for predefined routines, they lack flexibility when target configurations vary. Goal-conditioned manipulation policies address this limitation by conditioning actions on both the current cloth state and a desired goal state, such that the predicted actions transform the cloth toward the goal. Weng et al. (2022) propose FabricFlowNet (FFN), a dual-arm goal-conditioned policy for cloth folding that leverages optical-flow prediction, (Figure 7). Instead of predicting actions directly from the current and goal images, FFN decomposes the policy into two components: a FlowNet that estimates particle flow between the current observation and the goal, and a PickNet that predicts P&P points from the estimated flow image. Mo et al. (2022) introduce Foldsformer, which incorporates space-time attention (Bertasius et al., 2021) into a folding planner. Given the current cloth image and a sequence of demonstration images, the model outputs a sequence of multi-step action points. This design balances speed and accuracy while capturing manipulation points and their ordering, even when cloth pose and size differ from those in the demonstration.

#### 3.7.3 Predictive and model-based state representation methods

Goal-conditioned policies typically react to the current observation and goal, but do not explicitly model cloth dynamics or future evolution. Predictive and model-based methods aim to address this limitation by learning latent state representations or forward models of cloth behavior for planning and control. Hoque et al. (2022a) develop the VisuoSpatial Foresight (VSF) policy, trained on self-supervised simulated cloth manipulation data. VSF is built on Stochastic Variational Video Prediction (SV2P) (Babaeizadeh et al., 2018), an action-conditioned latent-variable video prediction model. At test time, the model receives the current and goal cloth states and predicts intermediate frames together with P&P coordinates, providing a visuospatial predictive model that can be used for planning. Ma et al. (2022) argue that human-defined labeled keypoints do not generalize well to unseen cloth configurations. They therefore use Transporter Networks (Kulkarni et al., 2019) to extract features and detect keypoints in an unsupervised manner from depth images. The detected keypoints are composed into a graph, and graph neural networks (GNNs) and recurrent networks are then used to model cloth dynamics in this learned space. Ganapathi et al. (2021) learn dense visual correspondences between different cloth configurations by training a Siamese network on pairs of cloth images, thereby capturing the underlying geometric structure. The learned correspondence field is used to transfer manipulation actions from a reference demonstration to new cloth states and has shown promising Sim2Real performance. Lin et al. (2022) propose a model-based RL approach in which a particle-based cloth dynamics model is learned from partial point clouds. A GNN models visible connectivity by operating on voxelized point clouds V and inferred edges E , and the learned dynamics are then used to train a P&P manipulation policy.

#### 3.7.4 Reward-driven reinforcement learning over primitive actions

**FIGURE 8:**
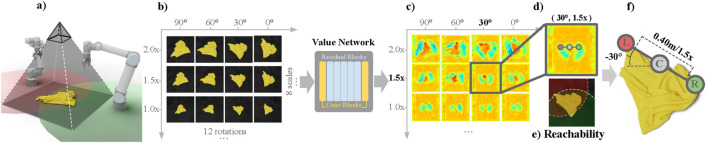
Use of Spatial Action Maps (Wu J. et al., 2020) in dynamic cloth actions (PMLR image credit: Ha and Song (2022), licensed under CC BY). **(a)** Workspace **(b)** Rotated and scaled images **(c)** Predicted value maps **(d)** Highest value **(e)** Reachability **(f)** Fling action.

Rather than explicitly modeling cloth dynamics, reward-driven RL acquires manipulation strategies by optimizing task-specific reward functions through trial-and-error interaction. This paradigm learns the parameters of primitive actions (e.g., pick, drag, or fling) autonomously, but typically requires careful reward design and large amounts of interaction data. Wu Y. et al. (2020) introduce an RL framework for P&P cloth unfolding in which the placing policy is learned conditioned on random pick points. The final pick location is then chosen as the point with maximal value under the learned placing policy (the maximum-value-under-placing, MVP, strategy), leading to faster learning than jointly learning pick and place. Ha and Song (2022) employ Spatial Action Maps (Wu J. et al., 2020) for dynamic cloth manipulation. Their method evaluates a batch of candidate fling actions by transforming the observation and predicting a batch of value maps (Figure 8). The pixel with maximal value that also satisfies reachability constraints is selected, and its location and transformation are decoded into fling parameters. This value-map paradigm has been widely adopted in subsequent work on dynamic cloth manipulation, including He et al. (2023), Canberk et al. (2023), Gu et al. (2024), and Xu et al. (2022). Xu et al. (2022) improve the fling grasping strategy of Ha and Song (2022) by introducing edge-coincident grasp parameterizations to boost performance. Canberk et al. (2023) propose a factorized value-prediction model with two Spatial Action Maps networks (each with one encoder and two decoders) to generalize value maps over two primitive actions. Gu et al. (2024) apply this factorized policy to cloth unfolding and augment the value maps with an additional detection module. Lee et al. (2021) adopt an offline, batch-RL setting: a real robot first collects data via random actions, and a DQN is then trained on this fixed dataset, with DA used to improve robustness in low-data regimes. To scale reward-driven RL, Ha and Song (2022) further propose a self-supervised interaction framework in simulation: the robot interactively unfolds cloth, and the simulator computes coverage after each action. Episodes are reset once coverage reaches a threshold or an action limit is exceeded, eliminating the need for expert demonstrations or ground-truth state labels. Avigal et al. (2022) extend this idea to the real world by using a small set of human-labeled primitives and gripper poses for initial training, followed by large-scale self-supervised data collection.

#### 3.7.5 Demonstration-driven skill transfer methods

Reward-driven RL can be sample-inefficient and sensitive to reward design, especially in real-world cloth manipulation. Demonstration-driven methods mitigate these challenges by leveraging expert demonstrations to provide more structured supervision. Seita et al. (2020) introduce behavior cloning (BC) for cloth unfolding. An Oracle supervisor generates unfolding demonstrations in simulation, and the policy is trained to imitate the supervisor from observed states. To improve robustness outside the demonstration distribution, they employ Dataset Aggregation (DAgger) (Ross et al., 2011), relabeling states visited under the learned policy, while DR over cloth appearance and camera poses supports Sim2Real transfer. Fu T. et al. (2023) propose a BC-based human-to-robot skill transfer framework for cloth unfolding. They decompose demonstrations into policy demonstrations (human-chosen P&P points) and action demonstrations (human manipulation trajectories), and use a mixture density network with parameter weighting to handle the multi-modal nature of unfolding behavior. The learned policy successfully unfolds cloth of various colors and sizes in the real world, with performance comparable to human operators. Lee et al. (2024) learn cloth manipulation actions directly from a small set of human videos (15 annotated demonstrations) to handle both unfolding and folding. A unified P&P policy is trained from these videos and deployed on a real robot, generalizing across fabrics with different shapes, colors, and textures. Other work, such as Goal-Aware GAIL (Tsurumine and Matsubara, 2022), explores adversarial imitation learning without hand-designed reward functions, but adversarial IL remains less common in current cloth manipulation studies.

#### 3.7.6 LLM-based planning methods

While previous paradigms focus on learning low-level perception or control policies, they typically lack high-level semantic reasoning and task abstraction. LLM-based planning methods address this gap by leveraging large language or multimodal models to perform high-level decision making over manipulation primitives. Fu et al. (2024) introduces a large language model into cloth unfolding. They prompt ChatGPT with task requirements, a predefined taxonomy of cloth states, and corresponding operational primitives. The LLM then recommends which primitive to execute next. A segmentation network subsequently identifies manipulation points for the chosen primitive, combining LLM-based decision making with visual perception. Raval et al. (2024) first detects cloth corners using a perception module and converts them into structured representations, which, together with human instruction prompts, are provided to ChatGPT for high-level reasoning to determine P&P points for the robot. In addition, an unselected study, Deng et al. (2025), leverages vision-language models to predict manipulation plans from visual observations and semantic keypoints, whereas earlier approaches rely solely on text-based inputs.

## 4 Discussion

As detailed in the results section, we analyze the key factors related to learning-based approaches for cloth manipulation and summarize their applications across unfolding and folding tasks. In this discussion, we further examine the current state, challenges, and perspectives associated with these factors, as outlined in Table 5, and highlight the strengths, limitations, and future opportunities of contemporary learning paradigms in this domain.

**TABLE 5:** Perspectives and opportunities for the factors that influence cloth unfolding and folding.

Keywords	Current state/challenges	Perspectives and opportunities
Task contents	• Unfolding and folding are treated as separate tasks • Limited cloth types (mainly towels/garments)	• Integrate unfolding and folding into a unified end2end pipeline • Improve generalization across diverse cloth types • Address complex cases (e.g., sleeves inside garments, inside-out T-shirts)
Dataset	• Simulation data dominate, while simulators lack realism and usability	• Develop more realistic, user-friendly cloth simulators (textile-air interactions, better control, diverse models) • Use VR or human-in-the-loop simulation to reduce the Sim2Real gap
	• Various Sim2Real methods exist but with inconsistent transferability	• Combine multiple Sim2Real strategies for improved robustness • Balance simulation and real-world transfer (RGB-D excels in simulation; depth transfers better) • When fine-tuning on real-world data, consider supervision signal availability and diversity
	• Real-world data collection is labor intensive and sensor limited	• Improve the convenience and richness of real-world data collection • Develop real-time human-intervention systems • Incorporate diverse sensing modalities • Leverage human demonstrations and online manipulation videos
Manipulation platform	• Platforms include single, dual, and triple arm setups	• Choose arm configurations by strategy and usability; dual arms support complex tasks
	• Grippers are mainly parallel types	• Multi-fingered hands could enable richer and more dexterous manipulation
	• Sensors are mostly vision-only, calibration is often required	• Choose RGB, depth, or RGB-D based on task properties • Incorporate multi-modal sensing (joint positions, force, tactile)
Primitive action	• Primitive actions continue to evolve	• Develop new primitive actions via improved tools, strategies, and learning-based policies
Performance metrics	• Unfolding metrics are relatively standardized, while folding lacks unified evaluation criteria	• Employ multiple complementary evaluation metrics • Establish a consensus metric for folding tasks
Failure modes	• Many failure modes (prediction errors, grasp failures, multi-layer grasping)	• Reduce prediction errors and Sim2Real gap • Improve platform or add sensors to prevent grasp failures • Consider richer grasp parameters (position, orientation, velocity)

### 4.1 Inferences drawn from the manipulation tasks

The unfolding and folding tasks are often studied separately. As discussed in Section 3.1, most papers focus exclusively on either folding or unfolding, with an equal distribution between the two topics. For those interested in exploring both tasks simultaneously, it is typical to propose either two separate policies or a single policy with distinct training for each task. However, separate unfolding and folding models require duplicating learning processes, which can be inefficient in terms of both computation and data usage. Therefore, integrating separate unfolding and folding policies into a unified end2end learning policy is a promising research direction. Only a few research papers (Xue et al., 2023; Lee et al., 2024) address this approach. A unified end2end learning policy is designed to manipulate cloth from a random initial state to a folded result, without focusing on the intermediate flattened state. This integrated policy requires training only once to handle the entire pipeline, eliminating the need for separate policies for unfolding and folding. This approach can reduce computational resources and deployment overhead, making the system more efficient. Additionally, the integrated policy may offer improved generalization capabilities.

When manipulating objects, using a common-sized towel is a regular choice, as it simplifies the problem. However, this policy may fail in some special circumstances where larger or everyday garments are involved. To address this, several studies have incorporated larger clothing or everyday garments into their research, effectively tackling related issues. These shifts in focus have led to further advancements. For example, the use of dynamic actions (Ha and Song, 2022) has facilitated the manipulation of larger cloths, while novel simulations (Lin et al., 2021) with diverse cloth models (Bertiche et al., 2020) have enabled handling a variety of garments. Despite the advancements made, more complex scenarios in cloth unfolding and folding remain. For instance, managing the sleeves or collar of a T-shirt, especially a long-sleeved one, can be challenging when the sleeve is tucked inside the garment. Additionally, when the entire T-shirt is inside-out, it further complicates the unfolding and folding tasks. These issues are common in daily life and need to be addressed in future research.

### 4.2 Issues and prospects for the dataset work

For DL-based methods, the dataset plays a crucial role. Section 3.2 highlights the primary methods of dataset collection from simulations and real-world data. While there are several advantages to using simulation, the simulators currently in use can only partially meet user needs. Therefore, a more realistic, convenient, and comprehensive clothing simulator is still needed. The ideal simulator should not only be able to load a wider variety of cloth models, simulate more realistic cloth textures, and provide a convenient API for users, but it should also offer aerodynamic interactions between air and cloth, which are essential for dynamic manipulation actions. Furthermore, the simulator could load the URDF of different robots to provide more detailed information about the interaction between the cloth and the robot.

Concerning the Sim2Real, the eligible papers have employed various solutions to mitigate this problem. The review finds that multiple Sim2Real technologies can be employed within a single research paper to enhance performance. However, in some cases, certain Sim2Real technologies may not be applicable due to limitations in the real-world setup. For example, Canberk et al. (2023) were unable to use the fine-tuning method due to the lack of available supervision signals in the real world. Despite this challenge, exploring this area remains a promising direction for future research.

In terms of the realworld dataset collection, current cloth keypoint collection methods included color painting labeling and UV labeling. Color painting is suitable for cases with various types of keypoints and requires only depth information for training. In contrast, UV labeling is better suited for scenarios that use RGB or RGB-D training datasets. However, it involves a limited number of keypoint types because there are few types of transparent UV fluorescent paint. Therefore, a novel keypoint labelling method is still required. Regarding data collection on robot and cloth interactions, current methods (Hoque et al., 2022b; Avigal et al., 2022; Lee et al., 2021) lack real-time feedback during dataset collection. There is a need for more convenient data collection methods that incorporate additional real-world information. One potential solution is the use of real-time feedback control platforms, such as ALOHA (Zhao et al., 2023), TactileAloha (Gu et al., 2025) and Gello (Wu et al., 2023), where a master robot is controlled by a human operator, while a slave robot performs the same actions in real-time to manipulate the cloth, thus collecting a more precise real dataset. Another approach involves using motion-capture systems, allowing a human operator to control the robot and manipulate the cloth, providing an intuitive and interactive method for dataset collection. Additionally, employing diverse sensors, such as force and tactile sensors (Kutsuzawa and Hayashibe, 2025; Gu et al., 2025), can further enhance the gathering of real-world information. Lastly, detecting the movement of human manipulation in videos is also a promising method because it is highly efficient and can utilize the considerable amount of video content on human manipulation available on the Internet. However, we must also address the gap between human manipulation and robot manipulation.

### 4.3 Implications of cloth manipulation platforms

Robotic platforms used for cloth manipulation vary widely in their mechanical capabilities and directly influence the design of learning algorithms. Single-arm systems (6–7 DoF) support basic P&P or one-arm fling motions (Chen et al., 2022), but generally exhibit limited versatility compared to dual-arm robots, which offer 12–14 DoF and enable coordinated bimanual strategies. Although triple-arm systems have been explored (Xu et al., 2022), they remain rare and are typically motivated by specialized manipulation or safety requirements. Overall, dual-arm configurations remain the most practical and capable choice for complex cloth tasks, despite their increased control and training complexity.

Across the reviewed papers, UR and Franka robots account for the majority of real-world deployments due to their user-friendly APIs, reliable hardware, and workspace geometries well aligned with cloth manipulation. Parallel grippers remain the predominant end-effector type; while dexterous (multi-fingered) hands promise richer manipulation behaviors, their high-dimensional control greatly increases algorithmic complexity and has limited their adoption.

For perception, RGB-D sensors remain the most widely used in cloth manipulation. However, visual observations sometime require preprocessing, e.g., segmentation or background removal (Mo et al., 2022; Tanaka et al., 2021), and different modalities (RGB, depth, RGB-D) can lead to noticeably different performance. Accurate calibration among robot, camera, and workspace frames is essential for Cartesian control (Ha and Song, 2022; Xu et al., 2022; Avigal et al., 2022; Canberk et al., 2023; Gu et al., 2024).

Beyond vision, multimodal sensing offers an underexplored opportunity for increasing robustness. Force and tactile feedback can mitigate common issues such as multi-layer grasping (Tirumala et al., 2022) or corner localization (Proesmans et al., 2023). Future systems will likely benefit from integrating such modalities, enabling more reliable grasping, improved perception under occlusion, and safer execution during dynamic actions.

### 4.4 Insights into the evolution of primitive actions

Primitive actions form the fundamental building blocks of cloth manipulation strategies, and their evolution reflects increasing requirements for precision, efficiency, and robustness. Traditional P&P primitives have been extended with parameterized or closed-loop variants (Hoque et al., 2022a; Blanco-Mulero et al., 2023; Hietala et al., 2022) to improve accuracy and adaptability. Dynamic fling actions (Ha and Song, 2022) dramatically accelerate unfolding and enable manipulation of larger garments, while follow-up work (Gu et al., 2024; Chen et al., 2022) further refines fling height, speed, and trajectory to improve reliability.

Additional primitives such as dragging or mopping (Xue et al., 2023; Avigal et al., 2022) expand the manipulation space by leveraging surface friction for local smoothing or global pose adjustment. Non-contact primitives such as air-blowing (Xu et al., 2022) demonstrate how external forces can unfold large surfaces safely and efficiently.

These developments illustrate that primitive actions are becoming increasingly specialized, combining coarse global adjustments with fine-grained corrections. As richer sensing modalities emerge, new primitives are likely to follow. For example, tactile-guided sliding (Sunil et al., 2023) enables reliable corner acquisition by using contact information to guide motion, a key step for both unfolding and folding. Going forward, learning frameworks will need to support hierarchical, multi-primitive, or hybrid controllers to take full advantage of this growing action diversity.

### 4.5 Performance metrics and failure modes to be concerned

Regarding performance metrics, the cloth unfolding task commonly employs two fundamental metrics: unfolded coverage and the number of manipulation actions. However, each eligible paper may introduce additional metrics tailored to their specific objectives, highlighting the performance of their proposed policies.

In contrast, there is no universally adopted basic metric for folding tasks, as each paper establishes its own criteria. While the folding success rate is frequently used, its definition varies across studies (Tsurumine and Matsubara, 2022; Deng et al., 2023). This lack of standardized metrics complicates the quantitative comparison of different approaches, particularly given the cloth’s deformability and the diverse range of experimental environments. To address these issues, researchers in this field should aim to report their baseline results and validation using a variety of metrics. This approach will facilitate comparisons with SOTA methods and enable a more comprehensive evaluation of their proposed approaches. On the other hand, a consensus metric for folding task also needs to be created in the future.

Moreover, some papers provide information on the failure modes encountered. These can be divided into two categories. The first category relates to the manipulation algorithm, including issues such as inaccurate predictions and the gap between simulation and reality. To address these issues, researchers should focus on improving algorithmic strategies. The second category involves factors not directly related to the algorithm, such as failed grasping or handling multiple layers. Solutions for these issues include improving platform settings, including more action parameters, or incorporating additional sensors. For instance, applying a nonslip silicone pad can increase grip friction, and grasp parameters should consider not only the coordinates but also the orientation of the grasp (Qian et al., 2020). Orientation information can reduce cloth deformation during grasping and help prevent failed grasps. Additionally, utilizing tactile sensors (Sunil et al., 2023) to detect layers can help avoid multiple-layer grasping.

### 4.6 Applications and opportunities of learning and control paradigms

As summarized in Section 3.7, the eligible papers can be reorganized into six learning and control paradigms that better reflect how existing methods perceive, represent, and act on cloth. Below, we discuss their advantage, disadvantage, and opportunities for cloth unfolding and folding, as outlined in Table 6.

**TABLE 6:** Advantages, disadvantages, and opportunities for the six learning and control paradigms.

Paradigm	Advantages	Disadvantages and opportunities
Perception-H	• Achieve accurate cloth perception using labeled data and strong vision backbones • Effective for structured folding tasks with explicit visual cues (e.g., corners, edges) • Modular and interpretable pipelines separating perception and control	•Require large-scale annotations; deformability and occlusion complicate labeling • Hand-crafted heuristics are brittle under topology changes, wrinkles and self-entanglement • Future work may replace fixed heuristics with learned controllers and extend to complex garments
Goal-cond	• Formulate manipulation as goal-reaching, naturally supporting multi-step folding • Enable data-efficient learning from goal images or collected trajectories • Capture spatiotemporal structure via attention or flow-based architectures	•Depend on well-defined goal states; ambiguous goals degrade performance • Often assume relatively neat initial configurations, limiting robustness • Promising directions include language- or semantic-goal conditioning and integration with planning or LLM-generated sub-goals
Predict.-model	• Learn explicit or latent cloth dynamics for look-ahead prediction and planning • Improve generalization via structured state representations (e.g., keypoints, correspondences) • Provide a principled interface between perception, control, and RL.	• Training dynamics models is computationally expensive and sensitive to model bias • Accuracy depends on simulator fidelity and scenario coverage • Opportunities include large-scale self-supervised learning and multimodal state fusion
Reward-driven	• Well suited for exploratory unfolding with highly variable initial states • Discover non-trivial strategies (e.g., dynamic fling) via trial and error • Avoid explicit labeling by relying on reward signals	• Require extensive interaction, which is costly in both simulation and real-world settings • Policies may suffer from Sim2Real gaps due to inaccurate simulator • Future work calls for better simulators, improved reward design, model-based/model-free RL, and offline or data-efficient RL.
Demo.-driven	• Learn policies from expert demonstrations without online interaction • Efficient for structured folding and routine-like tasks • Support diverse demonstration sources, including teleoperation and human videos	• Performance is limited by demonstration coverage and quality • Generalization across cloth types and materials remains challenging • Promising directions include scalable data collection, online correction, and multimodal (video, language) demonstrations
LLM-based	• Leverage semantic reasoning to select primitives and infer sub-goals • Multimodal LLMs enable visuomotor reasoning from visual inputs • Provide a unified interface for task specification, perception, and planning	• Current methods mainly operate at the high-level and lack low-level control • Inference latency limits real-time deployment on physical robots • Future work includes distilling high-level plans into lightweight controllers for real-time execution, integrating multimodal perception and action-generation models within low latency

#### 4.6.1 Perception-guided heuristic methods

Perception-guided heuristic methods are used for both unfolding and folding, particularly when some high-level geometric cues (e.g., corners, edges, contours) can be reliably extracted. By training detectors and segmentation networks on labeled cloth images (Seita et al., 2019; Gu et al., 2024), these approaches achieve high-accuracy perception and can directly localize manipulation-relevant regions on flattened or not severely wrinkled cloth, making them particularly suitable for simple unfolding and folding tasks. However, their reliance on hand-crafted post-processing and motion heuristics limits scalability. For example, downstream motion generation often ignores cloth deformability and contact dynamics, and heuristics tuned for one garment type or configuration frequently fail on heavily wrinkled, self-entangled, or topologically complex cloth, requiring manual redesign. In addition, the need for pixel-level labels and keypoints makes data collection expensive and restricts the diversity of cloth categories and configurations that can be covered. Future work could move from fixed geometric heuristics toward learned downstream controllers that take detector or segmentation outputs as input and are trained jointly with, or conditioned on, the follow-up actions. At the perception level, richer geometric and multimodal features (e.g., depth, 3D shape cues, or topological descriptors) and weaker forms of supervision could be used to reduce labeling costs. Extending these perception modules to operate robustly on non-flat, self-entangled garments and across a broader range of cloth categories would help perception-guided methods remain effective beyond narrowly structured folding scenarios.

#### 4.6.2 Goal-conditioned manipulation policies

Goal-conditioned manipulation policies frame cloth manipulation as a goal-reaching problem, mapping the current observation and a desired goal configuration to action sequences (Weng et al., 2022; Mo et al., 2022). This paradigm naturally aligns with folding tasks, whose target states are structured and can be expressed through goal images, keyframes, or demonstration trajectories. The primary challenge lies in goal specification and goal coverage. Existing approaches typically assume that a suitable goal image or trajectory is available and that the initial cloth state is not too far from this goal manifold. When the cloth is highly wrinkled, entangled, or heavily occluded, the system may struggle to infer a feasible goal-conditioned plan, and alternative paradigms (e.g., dynamics-based or RL-based methods) become more reliable. Future opportunities include allowing semantic goals (e.g., language descriptions, LLM-generated sub-goals) instead of explicit goal images, integrating predictive models to support longer-horizon reasoning, and learning goal manifolds that generalize across diverse garment categories and configurations. Such extensions would broaden the applicability of goal-conditioned policies beyond structured folding settings.

#### 4.6.3 Predictive and model-based state representation methods

Predictive and model-based approaches focus on learning cloth dynamics or latent state representations that support downstream planning and control (Hoque et al., 2022a; Ma et al., 2022; Ganapathi et al., 2021; Lin et al., 2022). A key advantage of this paradigm is reusability. Once an accurate dynamics or representation model is learned, it can support multiple tasks, unfolding, flattening, or different folding patterns, simply by changing the planner or objective, without retraining the entire policy. This separation of representation and control also improves data efficiency, since costly robot interaction is used to fit the model once, and later tasks can rely on planning or offline optimization over the learned dynamics. However, model-based methods face several challenges. Long-horizon cloth dynamics are difficult to learn: small prediction biases accumulate quickly and can mislead planning. Video prediction and GNN-based models require large and diverse datasets, yet often struggle to represent task-critical properties such as layer ordering, self-occlusion, or contact conditions. Self-supervised objectives focused only on reconstruction or local geometry may also fail to encode physically meaningful structures. Promising directions include learning representations that better capture cloth topology and layer structure; integrating learned dynamics with model-based RL or planning under uncertainty; and incorporating multimodal cues, such as depth, force, or tactile feedback, to resolve ambiguities in partially observed states.

#### 4.6.4 Reward-driven reinforcement learning over primitive actions

Reward-driven RL over primitive actions is currently the dominant paradigm for cloth unfolding (Wu Y. et al., 2020; Ha and Song, 2022; Canberk et al., 2023; Gu et al., 2024; Xu et al., 2022; Avigal et al., 2022). These methods optimize value or policy functions over discrete or continuous primitives using task-specific rewards. This paradigm is particularly well suited for cloth unfolding, because initial states are highly variable, heavily occluded, and partially observable. Such uncertainty naturally requires exploration and long-horizon decision-making, and RL agents can discover non-obvious sequences of pulls, flings, or drags that increase coverage even without an explicit cloth model. In practice, prior work has explored different ways to shape this learning process, for example by learning placement points from random picks (Wu Y. et al., 2020), designing multi-primitive policies that strategically select among several manipulation actions (Canberk et al., 2023), and tailoring reward functions to emphasize coverage (Ha and Song, 2022) or directional constraints in unfolding (Gu et al., 2024). Despite their strengths, RL methods face several challenges. Training in simulation is computationally expensive and strongly dependent on cloth and sensor fidelity, while large-scale real-world interaction is difficult to collect. Self-supervised interaction frameworks (Ha and Song, 2022; Avigal et al., 2022) reduce labeling effort, but significant Sim2Real gaps remain, and purely real-world training tends to be limited in scale and scenario diversity (Lee et al., 2021). Promising research directions include developing more realistic yet efficient cloth simulators, improving Sim2Real transfer methods, exploring more sample-efficient learning strategies, designing rewards that better capture cloth-specific manipulation objectives, and leveraging offline or data-driven RL methods capable of reusing large collections of prior trajectories.

#### 4.6.5 Demonstration-driven skill transfer methods

Demonstration-driven methods learn manipulation skills directly from expert demonstrations using behavioral cloning or related imitation-learning techniques (Seita et al., 2020; Fu T. et al., 2023; Lee et al., 2024; Tsurumine and Matsubara, 2022). In practice, such methods have been applied to both cloth unfolding and folding. For unfolding, data-driven policies can clone expert sequences of pulls, flings, or shakes that gradually increase coverage. For folding and other structured subtasks, where expert strategies (e.g., aligning corners, placing creases, or executing a fixed folding sequence) are relatively consistent, demonstration-driven methods are particularly effective. Recent works further show that demonstrations collected in simulation, from real robots, or even from human videos can be transferred to robotic policies, sometimes with only a small number of annotated trajectories (Seita et al., 2020; Lee et al., 2021; 2024). However, these approaches are fundamentally limited by demonstration coverage and distribution shift. Policies often fail when encountering states that are not represented in the demonstrations, or when manipulating garments with different sizes, materials, and shapes. Thus, both the quality and the diversity of demonstrations are critical for robust performance across unfolding and folding scenarios. Future opportunities include improving data-collection platforms (Zhao et al., 2023; Wu et al., 2023) to make it easier to gather large, diverse, and high-quality demonstrations; combining offline imitation with selective online correction (e.g., DAgger-style relabeling) to mitigate distribution shift; and systematically evaluating how different types of demonstrations and input modalities (simulation rollouts, real-world robot teleoperation, and human-hand videos) influence generalization to new garments, initial configurations, and task variations.

#### 4.6.6 LLM-, VLA-, and action-generation-based models

LLM-based planning for cloth manipulation is still in its early stage. Fu et al. (2024); Raval et al. (2024); Deng et al. (2025) illustrate a transition from text-only LLM prompting to multimodal inputs that include cloth observations for guiding high-level cloth manipulation planning. Recent multimodal LLMs such as Gemini (Team et al., 2025) further suggest the feasibility of mapping visual observations directly to manipulation trajectories. Related progress in robotics, e.g., large-scale vision-language-action (VLA) models such as RT-2 (Zitkovich et al., 2023) and OpenVLA (Kim et al., 2024), as well as action-generation architectures such as Action Chunking Transformer (ACT) (Zhao et al., 2023) and Π0.5 (Intelligence et al., 2025), which directly map images or language instructions to robot trajectories. Despite this promise, several challenges remain for cloth manipulation. First, collecting sufficiently diverse garment data at the scale required by LLMs and VLAs is difficult, and existing web-scale datasets contain little fine-grained deformable-object interaction. Second, inference latency and model size limit real-time deployment. Even models trained on specialized garment datasets are typically evaluated in quasi-static settings, where cloth deformation is slow and replanning frequency is low. Current action generators remain slower than conventional policy inference and often overlook cloth-specific dynamics such as self-occlusion, multilayer contact, and fast, large deformations, properties essential for dynamic interactions such as flinging, catching, or in-air regrasping. Promising directions therefore include finetuning or prompting LLMs and VLA models on cloth-manipulation datasets, distilling their plans into lightweight policies, and using them as high-level semantic planners that propose sub-goals or primitive sequences, while domain-specific controllers from the other paradigms (e.g., goal-conditioned policies, reward-driven RL, or demonstration-driven policies) execute those plans at a lower level and higher frequency. For action-generation architectures, a complementary avenue is to amortize expensive inference into compact latent plans or skill embeddings and let smaller, task-specific controllers decode short-horizon action chunks at control rate. This can both improve online speed and make such models more compatible with dynamic cloth primitives (e.g., fling or shake actions), enabling future systems to more fully exploit the flexible, highly deformable nature of garments rather than being limited to quasi-static operation.

## 5 Conclusion

This systematic review examined 41 deep learning-based robotic cloth unfolding and folding studies published between 2019 and 2024. From a systems perspective, we analyzed how task design, datasets, manipulation platforms, primitive actions, performance metrics, and failure modes jointly shape current solutions. From an algorithmic perspective, we reorganized the literature into six learning and control paradigms that more clearly reflect how cloth state is perceived, represented, and acted upon.

Across these works, several overarching insights emerge. First, most methods still treat unfolding and folding as isolated tasks with task-specific pipelines, despite their natural interdependence. Developing unified end2end policies that operate from highly crumpled states to folded configurations remains an underexplored but impactful direction. Second, data remains a core bottleneck: it calls for more realistic simulators and real-world pipelines that use modern teleoperation, motion capture, or multimodal sensing such as force and tactile feedback (Gu et al., 2025; Kutsuzawa and Hayashibe, 2025). Third, the review also highlights that the emergence of novel primitive actions contributes to the development of the field. Furthermore, performance metrics vary widely, and establishing a standardized metric is needed for future work. Depending on the failure mode, appropriate recovery solutions should be implemented.

Methodologically, each paradigm contributes differently to the field. Perception-guided heuristics offer high-accuracy perception and simple motion generation for simple unfolding and folding tasks, but rely on hand-crafted rules and generalize poorly when cloth exhibit complex or severe wrinkles. Future work includes replacing heuristics with learned controllers and extending perception to more diverse cloth. Goal-conditioned policies effectively drive folding when suitable goal images or trajectories are provided but struggle when initial states lie far from the goal manifold. Making goals more semantic and integrating predictive reasoning may improve robustness. Predictive and model-based representation methods provide reusable structure by learning cloth dynamics or latent states for downstream planning, yet remain limited by data demands and difficulty modeling long-horizon deformation and layer interactions. Advances in multimodal, topology-aware dynamics models are a key direction. Reward-driven reinforcement learning excels in highly variable, occluded unfolding scenarios requiring exploration and multi-steps credit assignment, but suffers from high sample complexity and Sim2Real gaps. Progress will depend on better simulators, improved reward design, and more effective Sim2Real strategies. Demonstration-driven skill transfer efficiently acquires folding and structured subtasks but relies heavily on demonstration coverage and diversity. Scalable data-collection pipelines and imitation schemes with selective online correction will be essential for broader generalization. Emerging large language model-based and action-generation models contribute high-level semantic planning, goal decomposition, and trajectory synthesis. Future efforts will focus on cloth-specific finetuning, improving inference time, and using them as high-level planners atop domain-specific low-level policies. Overall, while significant progress has been made in cloth unfolding and folding, ongoing research and innovation remain crucial for addressing the remaining challenges for future Physical AI.

## Data Availability

The original contributions presented in the study are included in the article/supplementary material, further inquiries can be directed to the corresponding author.
